# Gratitude and Emotional Intelligence as Protective Factors against Cyber-Aggression: Analysis of a Mediation Model

**DOI:** 10.3390/ijerph17124475

**Published:** 2020-06-22

**Authors:** María Teresa Chamizo-Nieto, Lourdes Rey, John Pellitteri

**Affiliations:** 1 Department of Personality, Evaluation and Psychological Treatment, Faculty of Psychology, University of Málaga, Campus de Teatinos s/n, 29071 Málaga, Spain; mtchamizo@uma.es; 2 Department of Educational & Community Programs, Queens College, City University of New York, New York, NY 11367, USA; John.Pellitteri@qc.cuny.edu

**Keywords:** gratitude, emotional intelligence, cyberbullying, protective factors, adolescents

## Abstract

Cyber-bullying is becoming an increasing school and health problem affecting adolescents worldwide. A number of studies have examined risk factors and protective factors in cyber-bullying situations and their consequences on the psychological well-being of adolescents. Gratitude and Emotional Intelligence (EI) are two personal resources that have been shown to have beneficial effects on the health and the social, personal and psychological functioning of young people. Nevertheless, little is known about these two variables in the context of cyber-bullying. The main purpose of this study was to examine the roles of gratitude and EI in cyber-aggression. Specifically, we hypothesised a mediational effect of gratitude in emotional intelligence-cyber-aggression link. A total of 1157 students aged 12–18 years (54.4% females) completed several questionnaires assessing gratitude (Gratitude Questionnaire; GQ-5), EI (Wong and Law’s Emotional Intelligence Scale; WLEIS-S) and cyber-bullying (European Cyberbullying Intervention Project Questionnaire; ECIPQ). The results showed expected significant associations between the studied variables. Moreover, the structural equation model analysis confirmed that EI dimensions were indirectly associated with cyber-aggression via gratitude, even when controlling for the effects of socio-demographic variables. These findings provide evidence on why those adolescents high in emotional intelligence are less aggressive in cyber-bullying context and suggest possibilities for gratitude interventions to reduce aggressive actions by electronic means among adolescents. The theoretical and practical implications are discussed.

## 1. Introduction

### 1.1. Cyber-Bullying

New technologies have changed the way people relate to each other, bringing advances in ease, speed and accessibility to global communication. In spite of these advantages, using the Internet and other electronic communication devices can have negative consequences, one of these being cyber-bullying. Cyber-bullying is defined as “an aggressive, intentional act carried out by a group or individual, using electronic forms of contact, repeatedly and over time against a victim who cannot easily defend him or herself” [1] (p. 376). It offers aggressors a number of advantages over traditional bullying methods, for instance, anonymity, an infinite audience and the ability to harm others without the need for physical contact [2,3].

In recent years, the prevalence of this problem has increased. According to a report by Save the Children [4] on violence in the digital environment, 39.65% of young Spanish people aged 18–20 years had suffered cyber-bullying. Moreover, 45.83% of cyber-aggressors were friends or partners of cyber-victims.

Serious negative consequences can arise from cyber-bullying. Both cyber-victims and cyber-aggressors show lower scores on life satisfaction and higher levels of loneliness, depressive symptomatology, perceived stress [5,6], social anxiety and negative self-concept [6] and are more likely to have attempted suicide [7,8]. Moreover, cyber-aggressors exhibit lower empathy, kindness and responsibility and more neuroticism, anti-social behaviour and school problems [9].

Given the high percentage of people who have been involved in cyber-bullying situations and the impact on victims’ health and psychological adjustment, a considerable number of studies have explored its associated risk and protective factors (e.g., [10]). On the one hand, previous literature has found several personal, family or contextual risk factors which are key predictor of cyber-aggression behaviours. For instance, as personal factors, studies have indicated that having been a cyber-victim [11], higher levels of aggression [9,12,13], lack of self-control [9] and lower self-awareness when it comes to experiencing and regulating emotions [14] are predictors of cyber-aggression. Likewise, the family context (i.e., positive family dynamic and parental style) seems to be a significant predictor of all forms of cyberbullying [15]. Moreover, contextual characteristics (e.g., education systems, cultural values or socio-economic status, among others) also are key factors involved in the country differences in cyber-bullying rates [16].

Finally, the literature also shows evidence on the mitigating effect of several contextual (e.g., positive school climate) and family (e.g., better family relations and more positive problem-solving style) protective factors on cyber-bullying perpetration [10,17]. Regarding personal resources, past research has highlighted the beneficial effects of emotional intelligence and gratitude on psychological adjustment, well-being and social relationships in adolescents (e.g., [18,19]), which suggests that these factors may also serve to prevent or reduce cyber-bullying.

### 1.2. Emotional Intelligence

EI is theoretically defined as a mental ability that allows us to perceive, identify, understand and manage our own emotions and those of others and to act upon them [20,21]. From a global a perspective, higher emotional skills are related to a greater life satisfaction [22], more prosocial behaviours and better psychological adjustment [23]. Considering the specific dimensions of EI, results have found that adolescents with high emotional understanding reported better coping with stress and more positive relationships, whereas, lower levels of emotional regulation were related to more social anxiety and more symptoms of stress [18].

Regarding aggression, evidence suggests that people with lower EI exhibit more aggressive behaviours [24] and score higher on aggression, hostility and anger [25]. Specifically, adolescents who reported less use and regulate their emotions are more involved in cyber-bullying actions [14]. Conversely, higher scores in emotional understanding reduce the likelihood of becoming a cyber-aggressor [12,15]. After running an EI programme for adolescents, Garaigordobil and Peña-Sarrionandia [26] found that developing emotional skills prevented violent behaviour and increased assertive social-conflict resolutions in this group. Likewise, the education programme for adolescents (PREDEMA), developed by Schoeps et al. [27], showed that training in emotional competence for adolescents reduced cyber-bullying and fostered subjective well-being. Thus, previous studies provide evidence on the protective role of the development of EI on cyber-bullying situations, reducing the likelihood of cyber-aggression behaviours.

### 1.3. Gratitude

Another protective factor that has been shown to promote adolescents’ health is gratitude. Gratitude has been described as the stable disposition of being aware of and responding to other people’s kindness [28] and of appreciating the good that there is in the world [29]. Several studies have shown the benefits of receiving and expressing gratitude to our well-being [30], life satisfaction (e.g., [31]) and social relationships (e.g., [32]).

For instance, gratitude has been found to correlate with more perceived social support [33], to foster prosocial behaviours in both the benefactor and others [34,35,36], to improve relations between classmates and teachers [37] and to predict greater social integration [38]. It has also been found to reduce behavioural aggression in response to provocation [39], the tendency to denigrate partners [40] and anti-social behaviour [19]. Nevertheless, to the best of our knowledge, there are a lack of studies analysing this personal resource in the context of cyber-bullying and specifically on cyber-aggression.

Although few studies have investigated the relationship between EI and gratitude, some scholars have showed that they are positively and significantly correlated [41,42] and even, that gratitude is predicted by emotional intelligence [41]. EI is one’s ability to process emotional information effectively perceiving, using, understanding and managing emotion as its core, and it is developed through learning and experience. Gratitude is one’s tendency to recognize and respond with grateful emotions to positive experiences [28]. According to McCullough et al. [28], gratitude is an emotion. Therefore, it is easily understood the relationship between EI and gratitude.

### 1.4. Why Considerer Gratitude as a Possible Mediator in the Emotional Intelligence-Cyber-Aggression Link?

Accordingly, given the empirical evidence showing a relationship between EI and cyber-aggression [26,27] and EI and gratitude [41], it is hypothesized that gratitude may also play a mediator role in the relationship between EI and cyber-aggression. For example, previous research on gratitude has highlighted the role of understanding one’s own and others emotions in predicting gratitude expression, suggesting that people who better perceive, express and understand their own and others emotions feel gratitude more frequently and in more domains of their lives [41]. As gratitude is a feeling toward other people’s kindness or appreciation of the good in the world, it could be suggested that individuals with high emotional skills tend to feel gratitude more frequently and intensely, which ultimately might reduce the intentions to perform cyber-aggressive behaviours. However, no research has been conducted to explore the underlying role of gratitude in the EI-cyber-aggression link.

### 1.5. The Present Study

In order to gain a better understanding of the associations between EI, gratitude and cyber-bullying perpetration in a relatively large sample of Spanish adolescents, this study had a twofold aim. Firstly, we wanted to examine the relationships between gratitude, EI and cyber-aggression. We expected to find a positive correlation between gratitude and EI, in accordance with earlier studies, and a negative association between these two resources and cyber-aggression. As mentioned above, there are theoretical and empirical reasons for thinking that individual differences in gratitude might mediate the EI-cyber-aggression link. Therefore, we explored a mediation mechanism between EI and cyber-aggression by gratitude. Based on the above findings, we expected to find that gratitude mediates the effects of EI dimensions on cyber-aggression. The higher ability to perceive, use, understand and manage emotion, the easier an individual might become grateful, which ultimately makes it less likely to commit cyber-aggressive actions.

## 2. Materials and Methods

### 2.1. Participants and Procedures

The sample consisted of 1157 adolescents (629 females, 527 males, and one did not give a gender) aged 12–18 years (*M*_age_ = 13.78, *SD* = 1.33) (two people did not report their age). They were recruited from five education centres in the south of Spain and were distributed across the 7th (26.1%), 8th (27.1%), 9th (22.3%) and 10th (24.5%) grades. The nationality of most of the participants was Spanish (97.1%).

The procedure we adopted was approved by the Ethical Committee of the University of Malaga (62-2016-H) and conformed with the Declaration of Helsinki [43]. It consisted of participants voluntarily and anonymously completing a battery of questionnaires as part of a larger project. First, we contacted several education centres in the province of Malaga, informing directors and board members about the objectives and methodology of the research. The centres that agreed to participate signed a consent form and informed their students’ parents of the study. Adolescents, whose parents signed the consent form (four education centres) or did not clearly refuse to participate in the study (one education centre), completed the battery of questionnaires during an hour in a tutorial class. Two researchers were present during this time to answer any questions that the adolescents had concerning the questionnaires.

### 2.2. Measures

Participants were asked to supply sociodemographic data (i.e., gender, age, nationality and study grades). The assessment instruments used were as follows:

Gratitude as a disposition was measured by Gratitude Questionnaire (GQ) [28]. We used the Spanish version for adolescents that comprises 5 items and showed good psychometric properties (Cronbach’s α for two samples was 0.74 and 0.77) [44]. Each item was answered on a seven-point Likert scale ranging from 1 (totally disagree) to 7 (totally agree). An example of item is “I have so much in life to be thankful for”. In the current study, Cronbach’s α was 0.77.

Emotional Intelligence was measured using Wong and Law’s Emotional Intelligence Scale (WLEIS) [45]. This scale assesses 16 items across four dimensions: other’s emotion appraisal (e.g., “I am a good observer of others’ emotions”), self-emotion appraisal (e.g., “I really understand what I feel”), regulation of emotion (e.g., “I am quite capable of controlling my own emotions”) and use of emotion (e.g., “I am a self-motivating person”). Answers are scored on a seven-point Likert scale ranging from 1 (totally disagree) to 7 (totally agree). We used the Spanish version of the scale, which has an internal consistency of 0.92 [46]. In the present research, Cronbach’s α was 0.88 for the total scale. The reliability of the dimensions was as follows: 0.74 (self-emotion appraisal), 0.73 (other’s emotion appraisal), 0.77 (use of emotion) and 0.77 (regulation of emotion).

Cyber-aggressive behaviours were measured using the cyber-bullying aggression subscale of the European Cyberbullying Intervention Project Questionnaire (ECIPQ) [47]. This subscale comprises 11 items assessing the frequency of performing cyber-bullying behaviours over the last two months, for instance, “I said nasty things about someone to other people either online or through text messages”. Adolescents answered each item on a five-point Likert scale ranging from 0 (never) to 4 (more times a week). We used the Spanish version with a Cronbach’s α of 0.88 for the cyber-aggression subscale [48]. The reliability of this subscale in the current study was 0.84.

### 2.3. Data Analysis 

The data analyses were performed using SPSS v24 and AMOS 25.0 program (IBM Corp., Armonk, NY, USA). First, we identified the descriptive statistics, calculated the internal consistency of the study variables and performed Pearson correlation analysis by SPSS v24. Therefore, we used dimensions of each construct as indicators of latent variables, where dimensions are sums of individual items. Therefore, we tested the measurement model to evaluate the extent to which each of the latent variables was represented by its individual items. On the condition that the measurement model was accepted, subsequently, the structural model was tested by the maximum likelihood estimation in AMOS 25.0 program. Hu and Bentler’s criteria [49] were applied to assess the goodness of fit of the model: Chi-square statistic/degree of freedom (χ2/df), root-mean-square error of approximation (RMSEA) and standardized root-mean-square residual (SRMR) values lower than 0.08 indicate an adequate fit, and comparative fit index (CFI) values of 0.95 or higher reflect a good fit.

## 3. Results

### 3.1. Descriptive Statistics and Correlations

Table 1 shows the means, standard deviations, internal consistencies and Pearson’s correlations for the study variables. All study measures were correlated in the expected direction. Cyber-aggression displayed a significant and negative correlation with gratitude and the dimensions of EI, whereas the two resources significantly and positively correlated with each other.

### 3.2. Structural Model

We tested four structural models using the maximum likelihood estimation for each dimension of EI. The four-mediation analysis for each EI dimension as predictors are shown in Table 2.

First, regarding self-emotion appraisal (SEA), the direct path coefficient to the criterion (cyber-aggression) in the absence of the mediator (gratitude) was significant (β = −0.02; *p* < 0.05). Next, a partially mediated model with a mediator (gratitude) between SEA and cyber-aggression revealed a good fit to the data: χ2/df = 1.759, RMSEA = 0.026, SRMR = 0.003 and CFI = 0.991.

Second, regarding others’ emotion appraisal (OEA), the direct path coefficient to the criterion (cyber-aggression) in the absence of the mediator (gratitude) was significant (β = −0.01; *p* = 0.28). Next, a partially mediated model with a mediator (gratitude) between SEA and cyber-aggression revealed a good fit to the data: χ2/df = 1.964, RMSEA = 0.029, SRMR = 0.011 and CFI = 0.989.

Third, regarding use of emotion (UOE), the direct path coefficient to the criterion (cyber-aggression) in the absence of the mediator (gratitude) was significant (β = −0.01; *p* = 0.11). Next, a partially mediated model with a mediator (gratitude) between SEA and cyber-aggression revealed a good fit to the data: χ2/df = 1.655, RMSEA = 0.024, SRMR = 0.009 and CFI = 0.993.

Finally, regarding regulation of emotion (ROE), the direct path coefficient to the criterion (cyber-aggression) in the absence of the mediator (gratitude) was significant (β = −0.01; *p* < 0.01). Next, a partially mediated model with a mediator (gratitude) between SEA and cyber-aggression revealed a good fit to the data: χ2/df = 1.698, RMSEA = 0.025, SRMR = 0.001 and CFI = 0.992.

### 3.3. Mediating Effect Testing

The mediating effect of gratitude was tested by the bootstrap estimation procedure in AMOS 25.0. Because the indirect effect estimates generally do not follow a normal distribution, a bootstrap sample of 5000 cases and at a 95 per cent confidential interval (CI) tested the mediating effect [50]. Table 2 shows the 95% bias-corrected CIs of the direct and indirect effects; no interval was overlapped with zero. Therefore, gratitude partially mediated the effect of SEA and ROE on cyber-aggression, and also completely mediated the effect of OEA and UOE on cyberaggression.

## 4. Discussion

The purpose of this research was to analyse the potential role of gratitude as a mediator in the link between EI dimensions and cyber-bullying perpetration. As far as we know, this is the first study to examine this mediational approach in a sample of Spanish adolescents aged 12–18 years. As mentioned earlier, although evidence exists for the explanatory power of EI and gratitude in predicting cyber-aggression, the way in which gratitude might influence the emotional intelligence-cyber-aggression link has never been examined.

With regard to our first aim, our findings are consistent with the previous literature showing a significant positive relation between both resources [36,41,51], while negative correlations were found between cyber-aggression and EI [5] and between cyber-aggression and gratitude. Thus, being a grateful adolescent was associated with higher self-reported competence in using and managing one’s emotions. Likewise, higher self-reported awareness of the positive things in life and developing better emotional skills was related to less frequently inflicting harm on others intentionally through the use of electronic devices.

With respect to the role of EI and gratitude on cyber-bullying, on the one hand, our results showed a higher level of EI predicts less cyber-aggressive behaviour, in accordance with earlier studies (e.g., [12]). Higher levels of stress and social anxiety, as well as lower levels of life satisfaction, have been found to predict more frequent cyber-bullying behaviour [6]. Therefore, helping adolescents to develop emotional competences could in turn help them to use more adaptive strategies to cope with daily situations; this could reduce their levels of stress [18] and improve their subjective well-being and life satisfaction [27] and therefore might, by implication, reduce the likelihood of them engaging in cyber-aggressive behaviour.

Moreover, in line with previous studies [39,40], our results also showed that gratitude led to less violent behaviour, in our case, in cyberspace, where people do not share a physical environment. Our finding suggests that cyber-aggressive behaviour is less likely in more grateful adolescents, possibly as a result of their greater appreciation and respect of others, both acquaintances and strangers, because grateful people show more empathy [39] and social worth [35,40] as well as a greater tendency to perform prosocial behaviours [36].

Consistent with mediational approach, we did find some support that gratitude is an underlying mechanism in the link between emotional intelligence and cyber-aggression in a sample of Spanish adolescents. This research suggests that gratitude mediated the influence of EI dimensions on cyber-aggression and that EI can positively predict gratitude. One plausible explanation for the association between EI and cyber-aggression is related to the state that people who report higher levels of self-emotion appraisal and other’s emotion appraisal and use emotions and ability to regulate emotions can become more grateful because they are more sensitive to the emotions, thoughts and actions that underlie the positive contributions of others than people who report lower levels of EI. An expected consequence is that grateful people behave more prosocially and will be less inclined to find reasons to become angry and aggressive [39]. However, future lines of research could formulate integrative models that consider dispositional, familiar, cultural and social dimensions, which would entail a fuller account of the cyber-bullying phenomenon during adolescence [52]. Hence, these findings suggest that beyond the indirect influence of emotional intelligence through gratitude, emotional intelligence has a direct impact on cyber-aggression in Spanish adolescents. Those adolescents with better emotional abilities have fewer negative emotions related to the expression of aggression or anger [42] due to the moderating role of EI [53]. Thus, the findings of the current research suggest that both gratitude and emotional intelligence are relevant protective factors for the prevention of becoming a cyber-aggressor.

The contributions of this research should be assessed in light of several limitations. First, in spite of the fact that the sample was large, our participants were from one specific region of Spain. This restricts our ability to extrapolate the findings to other geographical areas. Thus, in future research, it would be interesting to use a more heterogenous sample to confirm and generalise our results. This study focused on a Spanish adolescent population. Future research should explore whether gratitude accounts for the link between emotional intelligence and cyber-aggression in adolescents from other cultural groups (e.g., East Asians [54]). Additionally, because the research adopted a cross-sectional design, we must be cautious about drawing any causal inferences. Furthermore, we used only self-report measures, which may have introduced bias into participants’ responses. Using other measures in nationally representative samples from other countries would allow to generalise our findings and complement our results.

In spite of these limitations, the study has some important implications to consider. From a theoretical point of view, our results serve to increase our knowledge of protective factors in cyber-bullying situations, specifically the role of two personal resources (i.e., EI and gratitude). Likewise, our results give some preliminary support for the mediational model, contributing to a growing body of research that investigates how specific deficits in emotional skills might vary on aggressive behaviours due to the intervening role of developed positive or negative emotions in cyber-bullying situations. From a practical point of view, our findings highlight the importance of fostering EI as a personal resource that may be a potential way to foster positive climate at schools and reducing aggressive actions. Second, our study identified mediating variables that could potentially serve as the basis for school counselling interventions. As positive relationships appear to be an important aspect of school climate, thus reducing antisocial behaviour, school counsellors might increase grateful responding between adolescents by training them to notice the good in one’s life school and by encouraging positive appraisals that have been found to promote gratitude. Furthermore, EI skills training might also help adolescents to regulate their emotions and manage social interactions creating stronger relational bonds at school and ultimately reducing aggressive behaviours in online settings.

## 5. Conclusions

In sum, this study provides evidence of the underlying role of gratitude in the EI-cyber-aggression link. Moreover, our findings not only confirm the importance of gratitude as an individual characteristic that might reduce online violent behaviours but also suggest that anti-cyberbullying interventions aimed at increasing emotional skills and gratitude might be particularly useful for promoting positive peer functioning.

## Figures and Tables

**Table 1:** Descriptive statistics, internal consistency and correlations among variables.

Variables	α	Mean	SD	1	2	3	4	5	6
1. Cyber-aggression	0.84	0.16	0.33	-					
2. Gratitude	0.77	5.65	1.14	−0.19 **	-				
3. SEA	0.74	4.99	1.26	−0.14 **	0.39 **	-			
4. OEA	0.73	5.03	1.24	−0.08 **	0.30 **	0.43 **	-		
5. UOE	0.77	4.81	1.35	−0.12 **	0.43 **	0.55 **	0.40 **	-	
6. ROE	0.77	4.51	1.37	−0.13 **	0.32 **	0.61 **	0.32 **	0.57	-

Note: SEA, self-emotion appraisal; OEA, others’ emotion appraisal; UOE, use of emotion; ROE, regulation of emotion; SD, standard deviation. ** *p* < 0.01.

**Table 2:** The test of indirect effect in model of EI and cyber-aggression mediated by gratitude.

Model Pathways	Estimated Effect	SE	95 % Bias-Corrected CI		Z	*p*
			Lower	Upper		
Direct effect						
SEA → Cyber-aggression	−0.02	0.01	−0.03	−0.01	−2.42	0.016
SEA → Gratitude	0.34	0.02	0.28	0.40	12.05	<0.001
Gratitude → Cyber-aggression	−0.04	0.01	−0.06	−0.02	−4.00	<0.001
Indirect Effect						
SEA → Gratitude → Cyber-aggression	−0.01	0.01	−0.02	−0.01	−3.82	<0.001
Total Effect	−0.03	0.01	−0.05	−0.02	−4.19	<0.001
Direct effect						
OEA → Cyber-aggression	−0.01	0.01	−0.02	0.01	−1.07	0.285
OEA → Gratitude	0.26	0.02	0.21	0.32	9.62	<0.001
Gratitude → Cyber-aggression	−0.05	0.01	−0.07	−0.03	−4.46	<0.001
Indirect Effect						
OEA → Gratitude → Cyber-aggression	−0.01	0.01	−0.02	−0.01	−3.90	<0.001
Total Effect	−0.02	0.01	−0.04	−0.01	−2.54	0.011
Direct effect						
UOE → Cyber-aggression	−0.01	0.01	−0.03	0.01	−1.56	0.119
UOE → Gratitude	0.36	0.02	0.31	0.41	14.91	<0.001
Gratitude → Cyber-aggression	−0.04	0.01	−0.07	−0.02	−3.94	<0.001
Indirect Effect						
UOE → Gratitude → Cyber-aggression	−0.01	0.01	−0.02	−0.01	−3.72	<0.001
Total Effect	−0.03	0.01	−0.04	−0.01	−3.64	<0.001
Direct effect						
ROE → Cyber-aggression	−0.01	0.01	−0.03	−0.01	−3.00	0.003
ROE → Gratitude	0.26	0.02	0.21	0.31	9.94	<0.001
Gratitude → Cyber-aggression	−0.04	0.01	−0.07	−0.02	−3.97	<0.001
Indirect Effect						
ROE → Gratitude → Cyber-aggression	−0.01	0.01	−0.02	−0.01	−3.62	<0.001
Total Effect	−0.03	0.01	−0.04	−0.01	−4.90	<0.001

Note: Estimated using bias corrected and accelerated bootstrapping, with 5.000 samples. CI, confidence interval; SEA, self-emotion appraisal; OEA, others’ emotion appraisal; UOE, use of emotion; ROE, regulation of emotion.
